# Adaptation and validation of the Iowa infant feeding attitude scale and the breastfeeding knowledge questionnaire for use in an Ethiopian setting

**DOI:** 10.1186/s13006-020-00269-w

**Published:** 2020-04-09

**Authors:** Misra Abdulahi, Atle Fretheim, Alemayehu Argaw, Jeanette H. Magnus

**Affiliations:** 1 Department of Population and Family Health, Jimma University, Jimma, Ethiopia; 2 Department of Community Medicine and Global Health, University of Oslo, Oslo, Norway; 3 Norwegian Institute of Public Health, Oslo, Norway; 4 Department of Food Technology, Safety and Health, Faculty of Bioscience Engineering, Ghent University, Ghent, Belgium; 5 Faculty of Medicine, University of Oslo, Oslo, Norway; 6 Department of Global Community Health and Behavioral Sciences, Tulane School of Public Health and Tropical Medicine, New Orleans, USA

**Keywords:** Optimal breastfeeding, Knowledge, Attitude, IIFAS, Reliability, Validity, Developing country, Ethiopia

## Abstract

**Background:**

Validated instruments to assess breastfeeding knowledge and attitude are non-existent in Africa including Ethiopia. We aimed to adapt and validate the Breastfeeding Knowledge Questionnaire (BFKQ) and the Iowa Infant Feeding Attitude Scale (IIFAS) for use in Afan Oromo (AO), the most widely spoken language in Ethiopia.

**Methods:**

After forward-backward translation into Afan Oromo, the instruments were reviewed for content validity by a panel of a nutritionist and pediatricians, and pretested on a sample of 30 mothers. Then, a cross-sectional study involving 468 pregnant women in their second and third trimester was conducted between May and August 2017 in the Manna district, Southwest Ethiopia, using the final versions of the adapted questionnaires. We used exploratory and confirmatory factor analysis to assess the construct validity, receiver operating characteristic (ROC) curves to determine the predictive validity and Cronbach’s alpha coefficients to assess internal consistency.

**Results:**

Using exploratory factor analysis (EFA), nine domains containing 34 items were extracted from the BFKQ-AO. A confirmatory factor analysis of the constructs from EFA confirmed construct validity of the instrument (χ2/df = 2.11, RMSEA = 0.049, CFI = 0.845, TLI = 0.823). In factor analysis of the IIFAS, the first factor explained 19.7% of the total variance and the factor loadings and scree plot test suggested unidimensionality of the tool. Cronbach’s alpha was 0.79 for the BFKQ-AO and 0.72 for IIFAS-AO suggesting an acceptable internal consistency of both instruments. For the sensitivity and specificity in predicting intention of breastfeeding for ≥24 months, the area under the curve (AUC) was 82% for IIFAS score and 79% for BFKQ score.

**Conclusions:**

Here we present the first study that reported the use of the BFKQ and the IIFAS in Ethiopia. Our results showed that both BFKQ-AO and IIFAS-AO can be reliable and valid tools for measuring maternal breastfeeding knowledge and attitude in the study population, showing the potential for adapting these tools for application in a wider Ethiopian context.

## Background

Maternal knowledge and attitude towards optimal breastfeeding practices are factors that affect practices of breastfeeding in addition to socio-demographic factors [1–5]. Both knowledge and attitude are modifiable variables that can be addressed to improve breastfeeding practices [6–12]. Understanding maternal knowledge and attitudes toward breastfeeding hence guides the development and implementation of public health policy as well as evaluation of interventions aimed at increasing rates of breastfeeding. Therefore, valid and reliable instruments are required to assess knowledge and attitudes toward breastfeeding.

Adapting an existing instrument for a study has advantages of saving cost and time and involves fewer steps compared to developing a new instrument [13]. Moreover, a well-developed instrument with strong validity and reliability of the data in the source language as well as rigorously adapted and translated into several languages allows comparison of studies across cultures and languages. Furthermore, a field that utilizes existing instruments can build a knowledge base in which generalizations can be made and discussed across cultures in efforts to impact global public health. Therefore, for an instrument to be used outside the original setting, it has to be culturally adapted and its psychometric properties assessed [14].

The Iowa Infant Feeding Attitudes Scale (IIFAS) has been adapted and validated in several countries and demonstrated to have good predictive validity and excellent internal consistency ranging from a Cronbach’s alpha of 0.79 to 0.86 [15–28]. The Breastfeeding Knowledge Questionnaire (BFKQ) was developed for use in Malaysia [29] with a strong validity and reliability. On the other hand, to our knowledge, there are no breastfeeding knowledge and attitude instruments for which psychometric properties have been assessed in an African setting. Only one study in South Africa evaluated the content validity of IIFAS after cross-cultural adaptation though this was limited by the lack of a detailed psychometric assessment of the tool [30].Therefore, we aimed at adapting and validating the Afan Oromo versions of the BFKQ and the IIFAS among pregnant women in a rural setting from southwestern Ethiopia. The current study is part of the baseline study conducted for a Breastfeeding Education and Support Intervention (BFESI) trial [31].

## Methods

### Participants and study design

A cross-sectional study was conducted between May and August 2017 in Manna district under Jimma Zone, Southwest Ethiopia. From a total of 78 zones under Mana district, 36 study zones were selected based on geographic accessibility for study implementation. Pregnant women in the study zones were identified using the Antenatal Care log-book of the local Health Extension Workers [32]. Furthermore, we used leaders of the local Women Development Army groups to identify pregnant women not enrolled in the Antenatal Care [33]. All pregnant women (*n* = 468) in their second and third trimester living in the selected study zones, who met the study inclusion criteria were enrolled into the current study. Study inclusion criteria were pregnant women in their second and third trimester, without severe health complication including any psychiatric illness, who gave their consent to participate in the current study as well as in the subsequent BFESI trial with no plan to leave the study area before completion of the BFESI trial. Eligible participants were asked for their written consent of participation after they received an information session detailing the study, voluntary participation, and study withdrawal. Detailed description of the BFESI study setting is reported elsewhere [31].

### Study instruments

We adapted and evaluated Afan Oromo versions of two different instruments for assessing maternal knowledge on optimal breastfeeding (BFKQ-AO) and maternal attitude towards optimal breastfeeding practices (IIFAS-AO) for use in an Ethiopian setting. Permission to adapt and use the original versions of both IIFAS and BFKQ was obtained from the copyright holders Arlene De la Mora [34] and Tengku Alina Tengku Ismail [29], respectively.

### The Iowa infant feeding attitudes scale (IIFAS)

The IIFAS was [34] designed to assess maternal attitude towards infant feeding methods and to predict breastfeeding intention and exclusivity. The scale is composed of 17 items with a 5-point Likert scale ranging from 1 (strongly disagree) to 5 (strongly agree). The total IIFAS score can range from 17 to 85 with higher scores reflecting positive attitude towards breastfeeding. Total IIFAS scores can be further categorized into groups: 1) positive to breastfeeding (IIFAS score 70–85), 2) neutral (IIFAS score 49–69), and 3) positive to formula feeding (IIFAS score 17–48).

### The breastfeeding knowledge questionnaire (BFKQ)

The BFKQ, for assessing breastfeeding knowledge, was developed in Malaysia [29]. The tool contained a total of 47 questions covering different domains of knowledge on breastfeeding including advantages to mothers, advantages to babies, colostrum, effective feeding method, duration of breastfeeding, expressed breast milk, breast engorgement, problems with breastfeeding and practical aspects of breastfeeding. Each item has categorical responses of either ‘true’, ‘false’, or ‘not sure’. The BFKQ scores are converted into percentage scores using the denominator the possible maximum score for total BFKQ score and per knowledge domains.

### Instruments translation, content validity and administration

A systematic process recommended by Beaton et al. [14] was employed to develop the Afan Oromo versions of the instruments (IIFAS-AO and BFKQ-AO) while maintaining semantic, idiomatic, experiential and conceptual equivalence of the original English versions. Translation of instruments was carried out in a forward-backward procedure. The forward translation was conducted by bilingual professional translators with the help of the researcher (MA) who is a health professional. The backward translation to English from the Afan Oromo versions was carried out by two other professional translators who were totally blinded to the original English versions of the instruments. The original and the back-translated English versions of the instruments were compared to check for accuracy of the translation. We used a method developed by Skperber et al. [35] to establish semantic equivalence and validate the translated instruments. Each item in the original and back-translated instruments was ranked for comparability of language and similarity of interpretation. Ranking was done independently by 30 raters from academic members of Jimma University, Public Health Faculty using Likert scales ranging from 1 (reflecting extremely comparable/extremely similar) to7 (reflecting not at all comparable/not at all similar). Any item with a mean score of > 3 for comparability of language and/or a mean score > 2.5 for similarity of interpretability was considered problematic and required a formal review of the translation. In some items, problems were identified and corrected even if the mean scores were acceptable. Revision of problematic items followed the same procedure using a forward-backward translation followed by rating for comparability of language and similarity of interpretability until meeting the acceptable rating.

A team of experts composed of a nutritionist, two pediatricians, two professional translators and the first author conducted a qualitative review of the content validity of the Afan Oromo versions of the instruments for appropriateness in the study context. The instruments were then pilot tested among 30 pregnant women to assess its clarity, comprehension, length, and cultural acceptability. Based on feedbacks from the expert review and the pilot testing, a few modifications were made and the content validity of both instruments was finally confirmed.

### Psychometric analyses

For psychometric evaluation of the instruments, structured interview of participants was conducted to complete the two instruments and to gather data on other relevant variables including maternal intentions to breastfeeding, previous history of breastfeeding, socio-demographic, household, and maternal information. Data collection was carried out by ten nurses. They were trained for 2 days. Data was collected though a face-to-face interview after written consent was obtained from study participants. Two supervisors checked the completeness of filled questionnaires on daily basis.

Our sample size of 468 subjects was enough to validate the BFKQ-AO and IIFAS-AO tools with a total of 46 and 17 items, respectively considering the recommendation of 10 observations per variable for factor analysis [36]. Data were entered in duplicate using EpiData version 3.1 (EpiData Association) and consistency checks and statistical analyses were conducted using Stata version 13.1. Data were evaluated for normality by visual inspection of histograms and Q-Q plots and measures of kurtosis and skewedness, and expressed using mean (SD) or median (IQR) for the continuous variables and frequencies and percentages for the categorical variables. All statistical tests were two-sided with statistical significance considered at alpha < 0.05.

#### Construct (factorial) validity

A two-step approach of model building was carried out to develop a final version of the BFKQ-AO for the study context and evaluate its construct validity [37]. An iterative exploratory factor analysis (EFA) was carried out to extract the factors (latent variables) that fit the variance-covariance matrix of the observed variables. Principal components factor extraction method with Varimax rotation (Kaiser Normalization) was applied. A confirmatory factor analysis (CFA) was conducted in order to confirm the measurement model suggested by the EFA. Structural equation modeling (SEM) using maximum likelihood estimation was employed to assess the relationship between indicator variables and their corresponding latent variables and evaluate the overall performance of the CFA model. The need for any further model improvement like adding additional paths was examined using the modification of indices command. The overall fit of the model was evaluated by examining different goodness-of-fit statistics including the ratio of χ2 to degree of freedom (χ2/df < 3) [38], Root Mean Squared Error of Approximation (RMSEA < 0.06 ≤ 0.08) [39, 40], Comparative Fit Index (CFI ≥ 0.8 > 0.90) [40], Tucker-Lewis Index (TLI > 0.95) [41], Standardized Root Mean Square Residual (SRMR < 0.08) [40], Coefficient of Determination (CD) and the values of individual variable residuals.

Since the IIFAS items have been investigated in several previous studies in different contexts [15, 17–21, 24, 25, 28, 42] and the main goal at this stage was not item reduction, we applied criteria suggested by Nanishi et al. [24] to retain items in this analysis. Accordingly, invalid items were defined as 1) items with a negative loading to the first factor in factor analysis, 2) items that increased the alpha coefficient by > 0.10 when deleted, or 3) items that had a corrected item-total correlation of < 0.07. The later criterion was chosen based on the range of correlations (0.07–0.45) that were reported during the development of the original scale [23]. Prior to factor analysis, the suitability of our respondents’ data for factors extraction was examined using the Kaiser-Meyer-Olkin measure of sample adequacy (KMO > 0.5) and the Bartlett’s test of sphericity (*P*-value < 0.05).

#### Internal consistency reliability

Reliability of the instruments was determined by Cronbach’s alpha coefficient with alpha values ≥0.70 considered satisfactory [43]. Cronbach’s alpha values were calculated for the total scale of both IIFAS-AO and BFKQ-AO instruments and for the subscales of BFKQ-AO.

#### Predictive and criterion validity

The predictive validity of the total IIFAS-AO score and the total BFKQ-AO percentage score was examined by using the receiver operating characteristic (ROC) curves. The area under the graph was assessed for the sensitivity and specificity of both instruments in predicting mothers’ intention of breastfeed for at least 24 months. Regression analysis was carried out to identify the association between IIFAS-AO and BFKQ-AO. We used Pearson’s χ2-test to evaluate the association between demographic and socioeconomic variables and IIFAS-AO and BFKQ-AO scores.

## Results

### Sample characteristics

A total of 468 pregnant women were enrolled at baseline of the BFESI study [31]. The mean ± SD age of the women was 25.2 ± 4.96 years. All women were married 468 (100%) and the majority were housewives (93.8%). Nearly three forth (74.6%) of the women were illiterate. Eighty-six (18.4%) participants were primipara. All women intended to breastfeed (100%) and most of the women (84.4%) intended to breastfeed for ≥24 months (Table 1).

**Table 1:** Demographic and breastfeeding characteristics of pregnant women in Ethiopia (*n* = 468)

Variables	No.	%
**Age**		
< 20	50	10.7
20–34	390	83.3
35–40	28	6.0
Mean ± SD	25.2 (4.96)	
**Marital status**		
Married	468	100
**Educational status**		
Illiterate	349	74.6
Primary school	90	19.2
Secondary school	29	6.2
**Occupation**		
House wife/farmer	439	93.8
Other^a^	29	6.2
**Wealth index**		
Lowest	94	20.0
Second	94	20.0
Middle	93	20.0
Fourth	94	20.0
Highest	93	20.0
**Parity**		
Primiparous	86	18.4
Multiparous	382	81.6
**Gestational age**		
Mean ± SD	27.3	5.96
**Number of children (*n*** **= 370)**		
Mean ± SD	2.7	1.44
**Intention to breastfeed (n = 468)**		
Yes	468	100
**Intention to breastfeed for ≥ 24 months (n = 468)**		
Yes	395	84.4

^a^Government employee, merchant

### Psychometrics properties of instruments

#### Content validity

Based on the experts review and the pretest results, from the final 47 items in BFKQ in the Malaysian study, 2 items about keeping breast milk in refrigerator and 1 item about warming breast milk in microwave were dropped since these household items rarely exist in the rural part of Ethiopia. Moreover, 2 items about complementary feeding were excluded since they were out of the scope of the planned BFESI study. On the other hand, 4 additional items deemed important by the panel/experts, were added making a total of 46 items: 1 item in advantages to mother domain “Breastfeeding reduces bleeding that occurs after child birth.”, 1 in effective feeding domain “Correct attachment while breastfeeding helps accomplishing effective breastfeeding.” , 1 in breast milk expression domain “An expressed breast milk can stay up to 8 hours without getting spoiled.” and 1 in breast engorgement domain “It is possible to reduce breast engorgement with hot water.” . Items in the modified questionnaire covered the following scopes of knowledge on breastfeeding: advantages to mothers, advantage to babies, colostrum, effective feeding method, duration of feeding, expressed breast milk (EBM), storage of EBM, problems with breastfeeding and practical aspects of breastfeeding.

From the 17 IIFAS items, minor change were made in the three items: item number 4 “Breast milk is lacking in iron” was translated as “Breast milk doesn’t contain a mineral called iron which helps for blood production” since women had difficulty of understanding about the mineral ‘iron’; Item number 8 “Women should not breastfeed in public places such as restaurants” was translated as “Mothers should not breastfeed their child in public places e.g. wedding places, market places” as we do not have restaurants in the rural part of the region; Item number 16 “Breast milk is less expensive than formula” was translated as “Mother’s breast milk is cheaper than formula milk” to make it easy to understand.

#### Construct (factorial) validity

The Kaiser-Meyer-Olkin (KMO) measure of sampling adequacy and the Bartlett’s test of sphericity confirmed that our respondents’ data were suitable for factor analysis. The KMO values were 0.76 and 0.71 for the BFKQ-AO and IIFAS-AO data, respectively with KMO values > 0.5 for all items except items number 4 (KMO = 0.47) and 11 (KMO = 0.49) in the IIFAS scale. The Bartlett’s test of sphericity as well showed statistical significance for both scales (χ^2^ (df) = 3965 (561) & 1207 (136) for BFKQ-AO & IIFAS-AO, respectively; *P* < 0.001).

**Fig. 1:**
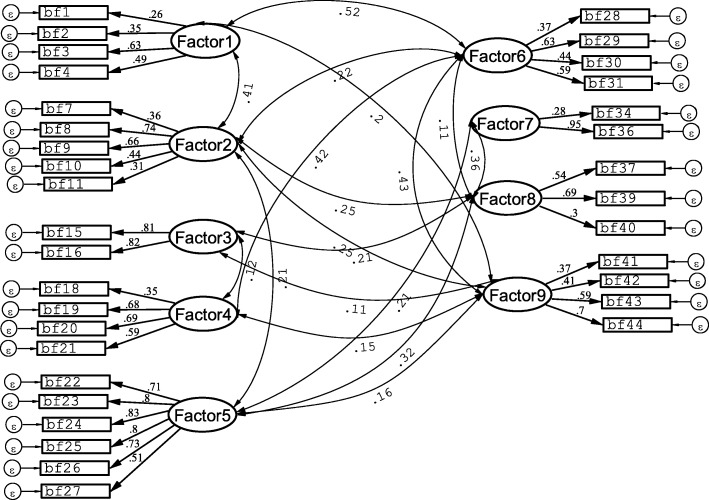
Path diagram for standardized parameter estimates of the BFKQ-AO measurement model using SEM (*n* = 468). The loading for each item is indicated by arrow. The lines between the factors show the correlation coefficients among nine factors. BFKQ-AO: Breastfeeding knowledge questionnaire-Afan Oromo. SEM: Structural Equation Modeling. Factor1, breastmilk expression; Factor2, advantages to mother; Factor3, effective feeding; Factor4, duration of feeding; Factor5, practical aspects of breastfeeding; Factor6, breast engorgement; Factor7, colostrum; Factor8, advantages to baby; Factor9, problem with breastfeeding

EFA using the initial BFKQ-AO with all 46 items yielded ten factors with Eigenvalues ranging from 1.04 to 4.77 and accounting for 58.1% of the total variance. We retained 34 items after dropping items with factor loading below 0.25, and also the tenth factor was not considered as an important construct because it did not contain positive loadings for most items and items with meaningful relationship. Thus, the final BFKQ-AO contained 34 items with 9 domains of breastfeeding knowledge. Table 2 shows the final items in BFKQ-AO and the factor loadings for their corresponding knowledge domains. A CFA of the nine domain BFKQ-AO suggested from the EFA showed an acceptable model fit including χ2/df = 2.11, RMSEA (95% CI) = 0.049 (0.045, 0.053), CFI = 0.845, TLI = 0.823, SRMR = 0.060 and CD = 0.999. Furthermore, all parameters for the association of items with their corresponding latent construct variable and the correlations among latent construct variables indicated in the model were statistically significant (*P* < 0.05) indicating the convergent validity of the measuring model (Fig. 1).

**Table 2:** BFKQ-AO items and their principal component factor loadings for corresponding domains

Domains	Items	Loadings
Advantages to baby (Factor 1)	Breastfeeding reduces the risk of lung infection among babies. (bf1)	0.48
	Breastfeeding increases the baby’s intelligence. (bf2)	0.71
	Breastfeeding helps to reduce the incidence of child abuse and neglect. (bf3)	0.63
	Baby who received breastfeeding is less prone to get diarrhea. (bf4)	0.40
Advantages to mother (Factor 2)	Exclusive breastfeeding is beneficial in spacing birth. (bf7)	0.50
	Breastfeeding helps to stimulate uterine contraction. (bf8)	0.79
	Breastfeeding reduces bleeding that occurs after childbirth. (bf9)	0.77
	Mothers who practised breastfeeding may achieve pre-pregnancy weight faster. (bf10)	0.48
	Frequent breastfeeding may prevent breast engorgement. (bf11)	0.29
Colostrum (Factor 3)	Colostrum is difficult to digest and needs to be discarded., median (IQR)* (bf15)	0.89
	Colostrum causes constipation among babies., median (IQR)* (bf16)	0.90
Effective feeding (Factor 4)	Babies will gain weight if they receive effective feeding. (bf18)	0.31
	Correct positioning helps to achieve effective breastfeeding. (bf19)	0.70
	Correct positioning helps to achieve effective breastfeeding. (bf20)	0.80
	Babies sleep well after they receive adequate breastfeeding. (bf21)	0.77
Breastmilk expression (Factor 5)	Breast milk expression may be done every 3 h. (bf22)	0.77
	An expressed breastmilk can stay up to 8 h without getting spoiled. (bf23)	0.83
	It is necessary to express breast milk from one side of the breast only.* (bf24)	0.85
	Expressed breast milk may be mixed with the previous expressed milk.* (bf25)	0.84
	Expressed breast milk may be warmed on a fire.* (bf26)	0.78
	The leftover expressed breast milk that has been used may be stored again.* (bf27)	0.59
Duration of feeding (Factor 6)	Breastfeeding should be initiated within 30 min after delivery. (bf28)	0.48
	Breastfeeding should be given on demand. (bf29)	0.67
	Baby should be allowed to breastfeed for at least 10–20 min for each fe. (bf30)	0.63
	Breastfeeding should be continued up to 2 years even though the baby has re. (bf31)	0.65
Problem with breastfeeding (Factor 7)	Breastfeeding must be discontinued if mother has cracked nipple.* (bf34)	0.67
	Breastfeeding must be discontinued if mother has breast engorgement.* (bf36)	0.69
Breast engorgement (Factor 8)	Breast engorgement may be reduced with cold packs. (bf37)	0.66
	The use of cabbage may help to reduce breast engorgement. (bf39)	0.76
	Massage may reduce breast engorgement. (bf40)	0.34
Practical aspects of breastfeeding (Factor 9)	Exclusive breastfeeding must be practiced until the infant is 6 months old. (bf41)	0.38
	Giving water to baby is encouraged after every breastfeeding.* (bf42)	0.35
	Belching after feeding shows that the baby is full. (bf43)	0.77
	Babies who get enough feeding will pass urine more frequently. (bf44)	0.73
		**overall**

*Reverse coded items. BFKQ-AO: Breastfeeding knowledge questionnaire-Afan Oromo

Extraction method: principal components analysis. Rotation method: Varimax with Kaiser Normalization. Twelve items were excluded since their loadings were < 0.25

**Fig. 2:**
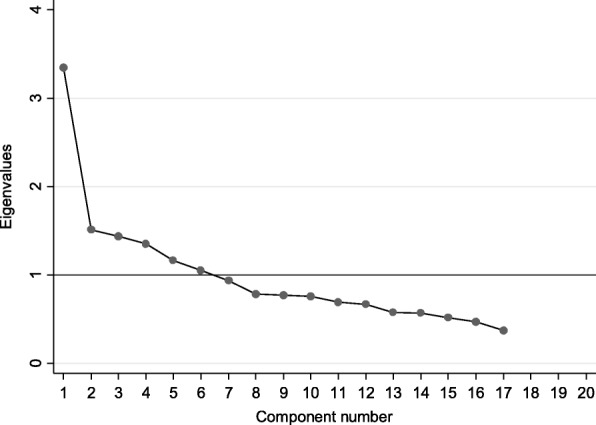
Scree plot of the 17-item IIFAS-AO scale with cut-off point for retained scale factors using Exploratory Factor Analysis (*n* = 468). IIFAS-AO: Iowa Infant Feeding Attitude Scale-Afan Oromo

Principal components factor extraction in the IIFAS-AO revealed that the scale has 6 components with Eigenvalues ranging between 1.06 and 3.35 and accounting for 58.1% of the variance. The first component with eigenvalue 3.35 accounted for 19.7% of the variance followed by the remaining 5 components with Eigenvalues ranging between 1.06 and 1.51 and with explained variance ranging between 6.21 and 8.88%. The scree plot, however, showed only one dominant factor suggesting that the scale is unidimensional (Fig. 2). The factor loadings for this first factor was positive and greater than 0.3 (range: 0.35–0.58) for all items except that items number 4 (0.16) and 11 (0.18) had a slightly lower loading (Table 3).

**Table 3:** IIFAS-AO items with means (SD), reliability results, and principal component factor loadings

Items	Mean (SD)	ρ	α^*^	Loading
1. The benefits of breastfeeding last only as long as the baby is breastfed.^**a**^	3.91 (1.08)	0.30	0.71	0.40
2. Formula feeding is more convenient than breastfeeding.^a^	3.83 (1.14)	0.30	0.71	0.42
3. Breastfeeding increases mother–infant bonding.	4.23 (0.78)	0.26	0.72	0.35
4. Breast milk is lacking in iron.^a^	3.00 (1.28)	0.13	0.73	0.16
5. Formula-fed babies are more likely to be overfed than breastfed babies.	3.62 (1.19)	0.41	0.70	0.53
6. Formula feeding is the better choice if the mother plans to go back to work.^a^	3.88 (1.05)	0.35	0.71	0.47
7. Mothers who formula feed miss one of the great joys of motherhood.	4.27 (0.78)	0.35	0.71	0.49
8. Women should not breastfeed in public places such as restaurants.^a^	3.73 (1.28)	0.45	0.70	0.58
9. Breastfed babies are healthier than formula-fed babies.	4.10 (0.92)	0.30	0.71	0.45
10. Breastfed babies are more likely to be overfed than formula-fed babies.^a^	3.93 (1.01)	0.34	0.71	0.45
11. Fathers feel left out if a mother breastfeeds.^a^	3.02 (1.19)	0.16	0.73	0.18
12. Breast milk is the ideal food for babies.	4.25 (0.82)	0.27	0.72	0.40
13. Breast milk is more easily digested than formula.	4.16 (0.86)	0.34	0.71	0.50
14. Formula is as healthy for an infant as breast milk.^a^	3.97 (0.97)	0.38	0.71	0.54
15. Breastfeeding is more convenient than formula.	3.95 (1.04)	0.37	0.71	0.51
16. Breast milk is cheaper than formula.	4.13 (0.89)	0.27	0.72	0.39
17. A mother who occasionally drinks alcohol should not breastfeed her baby.^a^	3.68 (1.27)	0.36	0.71	0.50
**Mean (SD) and Cronbach’s alpha (α) for total IIFAS-AO score**	**65.7 (7.64)**		**0.72**	

^a^Reverse-scored items. IIFAS-AO: Iowa Infant Feeding Attitude Scale-Afan Oromo

Abbreviations: α, Cronbach’s alpha for total IIFAS score based on the 17 attitude items; α*, Cronbach’s alpha if an item is removed; ρ, item-rest (corrected item-total) correlation for IIFAS items

### Internal consistency reliability

The participants had higher overall IIFAS score, with mean ± SD of 65.7 ± 7.6, ranging between 36 and 85, with the majority (60.9%) having a neutral attitude toward breastfeeding. Only 36.9% of participants had strongly positive attitudes toward breastfeeding. The Cronbach’s alpha for the IIFAS-AO was 0.72. The mean ± SD of each item, corrected item-total correlation, and alpha if item is removed from the scale are provided in Table 3. In total, all the 17 items were found important for the scale with alpha change if item removed not greater than 0.1 and the corrected item-total correlations were greater than 0.07 (range: 0.13–0.45) for all items.

The median percentage score for BFKQ-AO among the respondents was 76.5% (IQR 26.0). The Cronbach’s alpha scores and the median total and percentage scores for each domains of the BFKQ-AO are presented in Table 4. Cronbach’s alpha for the overall BFKQ-AO scale was 0.79, suggesting a good internal consistency reliability. With regard to reliability per domain, the Cronbach’s alpha coefficient was satisfactory (> 0.7) for breast milk expression and colostrum, moderate for advantage to mother and effective feeding and poor for advantage to baby, duration of feeding, problem with breastfeeding, breast engorgement and practical aspects of breastfeeding.

**Table 4:** Median (IQR) and Cronbach’s alpha for the BFKQ-AO scale

Subscales	No. of items	Median score (IQR)	Median percentage score (IQR)	Cronbach’s alpha
Advantages to baby	4	4.00 (3.00, 4.00)	100 (75.0, 100)	0.42
Advantages to mother	5	5.00 (4.00, 5.00)	100 (80.0, 100)	0.63
Colostrum	2	1.00 (0.00, 2.00)	50.0 (0.0, 100)	0.80
Effective feeding	4	4.00 (4.00, 4.00)	100 (100, 100)	0.62
Breast milk expression	6	4.00 (2.00, 4.00)	66.7 (33.3, 66.7)	0.87
Duration of feeding,	4	4.00 (3.00, 4.00)	100 (75.0, 100)	0.50
Problem with breastfeeding	2	0.00 (0.00, 1.00)	0.00 (0.00, 50.0)	0.41
Breast engorgement	3	2.00 (1.00, 3.00)	66.7 (33.3, 100)	0.50
Practical aspects of breastfeeding	4	3.00 (3.00, 3.00)	75.0 (75.0, 75.0)	0.51
Overall BFKQ-AO	34	26.0 (23.0, 27.0)	76.5 (67.6, 79.4)	0.79

*BFKQ-AO* Breastfeeding knowledge questionnaire-Afan Oromo; *IQR* Interquartile range

**Fig. 3:**
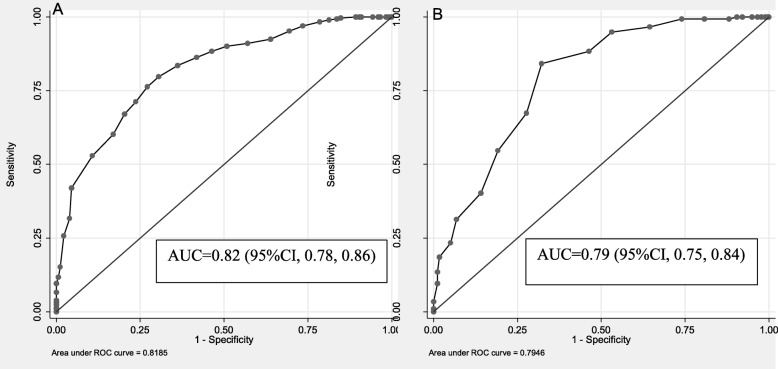
Area under the ROC curve for predicting pregnant women’s’ intention to breastfed for at least 24 months using the IIFAS-AO score (panel A) and BFKQ-AO score (panel B). ROC: Receiver operating characteristic curve. AUC: Area under curve. IIFAS-AO: Iowa Infant Feeding Attitude Scale-Afan Oromo. BFKQ-AO: Breastfeeding knowledge questionnaire-Afan Oromo

**Predictive and criterion validity.** In our sample, intention to breastfeed is universal. Thus, we used mothers’ intention of breastfeeding for at least 24 months to assess the predictive validity of the IIFAS-AO and BFKQ-AO. When the sensitivity and specificity of the total IIFAS score and BFKQ score were examined against intention of breastfeeding for ≥24 months, the area under the curve (AUC) for the ROC curve was 0.82 for IIFAS (95% CI, 0.78, 0.86) and 0.79 for BFKQ (95%CI, 0.75, 0.84) (Fig. 3). In univariate analysis, only maternal occupation was associated with BFKQ-AO (χ2 (df) = 4.99 (1); *P* = 0.026). None of the other demographic and socioeconomic variables showed association with IIFAS-AO and BFKQ-AO. However, IIFAS-AO was found to have significant association with BFKQ-AO (β = 0.33; 95% CI, 0.19–0.60).

## Discussion

To our knowledge, this is the first study reporting the adaptation and psychometric properties of the BFKQ and the IIFAS in Africa. The participants had high overall IIFAS-AO (65.7 ± 7.6) and BFKQ-AO score (76.5%). The 17 items IIFAS-AO and 34 items BFKQ-AO were found to have an acceptable level of internal consistency and reliability as confirmed by Cronbach’s alpha values > 0.70. The CFA showed that the BFKQ-AO has good construct validity. Factor analysis of the 17 items IIFAS also confirmed the unidimentionalty of the tool. Both the IIFAS-AO and the BFKQ-AO had good predictive validity for maternal intention of breastfeeding for ≥24 months.

In this study, the Cronbach’s alpha of IIFAS-AO was 0.72 which is acceptable for established tools [44], and is comparable to what has been reported for the original IIFAS tool with Cronbach’s alpha of 0.68. The corrected item-total correlations of 0.13–0.45 found in this study are also comparable to the original IIFAS of 0.07–0.45. Although the corrected item-total correlations for the 17 items were all positive and significant in this study, it was less than 0.30 for two items (items 4 and 11). This might be due to the fact that women living in rural part of Ethiopia may not have knowledge about the mineral iron and that they do not consider men as having any role in breastfeeding. However, these two items were kept in the IIFAS-AO because of their good alpha estimates and positive correlations.

When it comes to the BFKQ-AO, the overall Cronbach’s alpha at 0.79 was acceptable, and similar to the original Malay version of the questionnaire [29]. Moreover, in line with the original study, the overall median percentage score of BFKQ-AO was 76.5%. However, the percentage score was lower for domains of colostrum, breastmilk expression, problem with breastfeeding and breast engorgement. Moreover, even though the mean IIFAS score show that participants had positive attitude towards breastfeeding, attitudes about breastfeeding were more towards neutral to breastfeeding signifying the importance of targeted breastfeeding education to women.

The original Malay study assessed the construct validity of the BFKQ only using EFA. However, in the current study we confirmed the results obtained from EFA using CFA, which showed satisfactorily model fit for the domain constructs and convergence of the items in each factor/domain. Principal components factor extraction on the IIFAS-AO revealed that the scale has 6 components accounting for 58.1% of the variance. This finding is in-line with a study in Lebanon [15].

Many studies have assessed ability of IIFAS to predict intention to breastfeed [16, 18, 24, 42, 45–47]. However, in the current study, since all women had intention to breastfeed, we assessed whether IIFAS can predict intention for breastfeeding ≥24 months. Accordingly, the IIFAS-AO showed good predictive validity for mothers’ intention of breastfeeding ≥24 months. Although the original study of BFKQ did not assess the predictive validity, in this study we assessed it and found that the BFKQ-AO had good predictive validity for mothers’ intention of breastfeeding ≥24 months.

Regarding association between demographic and socioeconomic variables with the IIFAS-AO, in this study none of the demographic and socioeconomic variables were associated with IIFAS-AO. Contrary to this, other studies that assessed IIFAS report that age [15, 48], education [15, 25, 48], income/socioeconomic status [15, 25, 48], employment [25], parity [16], number of children [15], number of breastfed children [15], and duration of any breastfeeding were associated with IIFAS score. Only maternal occupation was found to have association with BFKQ-AO in the current study. Therefore, it is important to provide targeted breastfeeding education that focuses on improving knowledge and attitude towards breastfeeding to expecting mothers.

The strength of this study was that the sample size for factor analysis was adequate, the rigor related to the translation, the expert assessment of all items, the pilot of the cultural comprehensibility of the questions, and the comprehensiveness of the factor analyses of each instrument. This study also has some limitations: study participants were all married women from rural area with low educational status. Further studies are needed to evaluate the validity and reliability of BFKQ-AO and the IIFAS-AO in urban areas. Further longitudinal research is needed to assess the ability of the tools in predicting intention for early initiation and duration of exclusive breastfeeding.

## Conclusions

This is the first study to assess psychometric properties of IIFAS and confirmatory factor analysis of BFKQ in Africa. Over one third of all inhabitance in Ethiopia speak Afan Oromo. We found that both BFKQ-AO and the IIFAS-AO can be reliable and valid instruments for assessing maternal knowledge and attitude towards breastfeeding practice in the study population. The current study showed the potential of future translation, adaptation and use of these instruments for application in a wider Ethiopian context.

## Data Availability

The datasets used and/or analysed during the current study are available from the corresponding author on reasonable request.

## Abbreviations

BFESI: Breastfeeding Education and Support Intervention
BFKQ: Breastfeeding Knowledge Questionnaire
CFA: Confirmatory Factor Analysis
CD: Coefficient of Determination
CFI: Comparative Fit Index
EBF: Exclusive breastfeeding
EFA: Exploratory Factor Analysis
IIFAS: Iowa Infant Feeding Attitudes Scale
KMO: Kaiser-Meyer-Olkin
RMSEA: Root Mean Squared Error of Approximation
ROC: Receiver Operating Characteristic
SEM: Structural Equation Modelling
SRMR: Standardized Root Mean Square Residual
TLI: Tucker-Lewis Index
